# (*E*)-*N*-(Ferrocenyl­methyl­idene)(pyridin-3-yl)methanamine

**DOI:** 10.1107/S1600536811027346

**Published:** 2011-07-13

**Authors:** Nadia Adams, Thomas Gerber, Cedric McCleland, Richard Betz

**Affiliations:** a Nelson Mandela Metropolitan University, Summerstrand Campus, Department of Chemistry, University Way, Summerstrand, PO Box 77000, Port Elizabeth 6031, South Africa

## Abstract

In the title compound, [Fe(C_5_H_5_)(C_12_H_11_N_2_)], the cyclo­penta­dienyl rings are present in an eclipsed conformation. The imine is *E*-configured. In the crystal, C—H⋯N inter­actions involving both N atoms connect the mol­ecules into two undulating sheets perpendicular to the *b* axis. The centroid–centroid distance between the two aromatic systems in the ferrocenyl moiety is 3.2928 (18) Å. A C–H⋯π inter­action is also present.

## Related literature

For general background to ferrocenyl compounds, see: Nolan *et al.* (2007 ▶); Cheng *et al.* (2008 ▶); Quing *et al.* (2009 ▶); Bildstein *et al.* (1999 ▶). For graph-set analysis of hydrogen bonds, see: Etter *et al.* (1990 ▶); Bernstein *et al.* (1995 ▶).
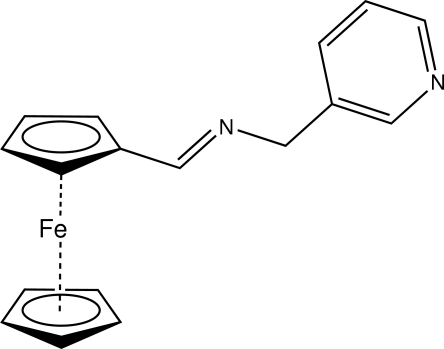


## Experimental

### 

#### Crystal data

[Fe(C_5_H_5_)(C_12_H_11_N_2_)]
*M* *_r_* = 304.17
Orthorhombic, 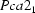

*a* = 18.7988 (17) Å
*b* = 5.9314 (6) Å
*c* = 12.5083 (10) Å
*V* = 1394.7 (2) Å^3^
*Z* = 4
Mo *K*α radiation
μ = 1.07 mm^−1^
*T* = 200 K
0.32 × 0.18 × 0.13 mm

#### Data collection

Bruker APEXII CCD diffractometer
6850 measured reflections
3226 independent reflections
2664 reflections with *I* > 2σ(*I*)
*R*_int_ = 0.059

#### Refinement

*R*[*F* ^2^ > 2σ(*F* ^2^)] = 0.033
*wR*(*F* ^2^) = 0.073
*S* = 0.94
3226 reflections
181 parameters
1 restraint
H-atom parameters constrained
Δρ_max_ = 0.63 e Å^−3^
Δρ_min_ = −0.34 e Å^−3^
Absolute structure: Flack (1983 ▶), 1437 Friedel pairs
Flack parameter: −0.010 (18)

### 

Data collection: *APEX2* (Bruker, 2010 ▶); cell refinement: *SAINT* (Bruker, 2010 ▶); data reduction: *SAINT*; program(s) used to solve structure: *SIR97* (Altomare *et al.*, 1999 ▶); program(s) used to refine structure: *SHELXL97* (Sheldrick, 2008 ▶); molecular graphics: *ORTEP-3* (Farrugia, 1997 ▶) and *Mercury* (Macrae *et al.*, 2008 ▶); software used to prepare material for publication: *SHELXL97* and *PLATON* (Spek, 2009 ▶).

## Supplementary Material

Crystal structure: contains datablock(s) I, global. DOI: 10.1107/S1600536811027346/vm2107sup1.cif

Supplementary material file. DOI: 10.1107/S1600536811027346/vm2107Isup2.cdx

Structure factors: contains datablock(s) I. DOI: 10.1107/S1600536811027346/vm2107Isup3.hkl

Additional supplementary materials:  crystallographic information; 3D view; checkCIF report

## Figures and Tables

**Table 1:** Hydrogen-bond geometry (Å, °) *Cg* is the centroid of the C21–C25 ring.

*D*—H⋯*A*	*D*—H	H⋯*A*	*D*⋯*A*	*D*—H⋯*A*
C13—H13⋯N2^i^	0.95	2.58	3.429 (4)	149
C34—H34⋯N1^ii^	0.95	2.59	3.482 (4)	156
C32—H32⋯*Cg*^iii^	0.95	2.92	3.775 (3)	150

Symmetry codes: (i) 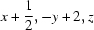; (ii) 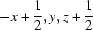; (iii) 
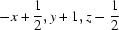
.

**Fig. 1.**
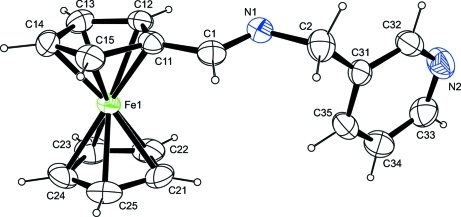
The molecular structure of the title compound, with atom labels and anisotropic displacement ellipsoids (drawn at 50% probability level).

**Fig. 2.**
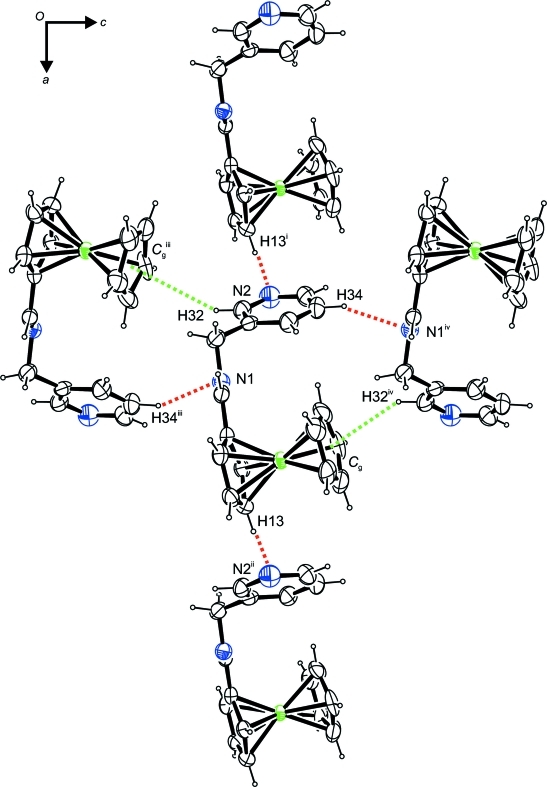
Intermolecular C–H···N contacts (red dashed lines) as well as C–H···π contacts (green dashed lines), viewed along [0 1 0]. Symmetry operators: ix - 1/2, -y + 2, z; iix + 1/2, -y + 2y, z; iii -x + 1/2, y, z - 1/2; iv -x + 1/2, y, z + 1/2.

**Fig. 3.**
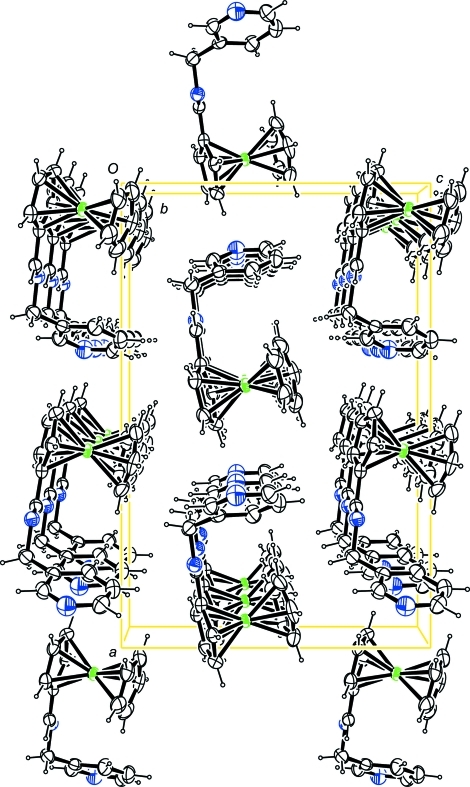
Molecular packing of the title compound, viewed along [0 - 1 0] (anisotropic displacement ellipsoids drawn at 50% probability level).

## supplementary crystallographic information

### Comment

Chelate ligands are an important class of molecules for complexation reactions. Given a specific set of donor atoms, their chelation ability in terms of Lewis basicity as well as denticity can be fine-tuned. Endowing the "backbone" of a chelate ligand with various substituents, a seemingly endless variety of ligands featuring different steric pretenses, solubility properties and derivatization possibilities is available. Ferrocenyl-substituted chelate ligands seem to be promising candidates with respect to all of the aforementioned features. In a larger study aimed at elucidating the rules guiding the formation and properties of *N*,*N*-ligand-supported coordination compounds, we determined the structure of the title compound to enable comparative studies. General background on ferrocenyl compound is amply available in the literature (Nolan *et al.*, 2007; Cheng *et al.*, 2008; Quing *et al.*, 2009; Bildstein *et al.*, 1999).

The cyclopentadienyl rings (Cp) in the ferrocenyl moiety are present in an ecliptic conformation. Both cyclopentadienyl moieties are nearly parallel to each other, the least-squares planes defined by their respective atoms enclose an angle of only 2.20 (14) °. The iron atom is a bit closer to the substituted Cp ring than to the unsubstituted one with the vertical displacements found at 1.6414 (5) Å and 1.6516 (5) Å. The imine is *E*-configured. The pyridyl system is roughly orientated perpendicular to the aromatic systems of the ferrocenyl moiety, the least-squares plane defined by its atoms intersects with the planes defined by the five-membered carbocycles at an angle of 78.66 (12) ° and 80.31 (11) °, respectively (Fig. 1).

In the crystal structure, two sets of C–H···N contacts are present whose ranges fall by about 0.1 Å below the sum of van-der-Waals radii of the atoms participating. While the N-atom in the pyridine-system acts as acceptor for one of the H-atoms of the substituted cyclopentadienyl-moiety, the second C–H···N contact is evident between the imine-type N-atom and the H-atom in *meta*-position to the N-atom in the pyridyl-moiety (Fig. 2). In terms of graph-set analysis (Etter *et al.*, 1990; Bernstein *et al.*, 1995), the descriptor for the first set of contacts is *C*^1^_1_(10) on the unitary level while the second set necessitates a *C*^1^_1_(6) descriptor on the same level. Additionally, a C–H···π interaction stemming from the CH group in *ortho* position to the nitrogen atom of the pyridine system is present. In total, the molecules are connected to wavy sheets perpendicular to the crystallographic *b*-axis (Fig. 2). The intercentroid distance between the two aromatic systems in the ferrocenyl moiety was measured at 3.2928 (18) Å.

The packing of the title compound is shown in Figure 3.

### Experimental

The title compound was prepared upon condensation of ferrocenyl aldehyde with (pyridin-3-yl)methanamine in an aprotic solvent. Crystals suitable for the X-ray diffraction study were obtained upon recrystallization from dichloromethane-*n*-hexane.

### Refinement

Carbon-bound H-atoms were placed in calculated positions (C—H 0.99 Å for the methylene group and C—H 0.95 Å for aromatic carbon atoms) and were included in the refinement in the riding model approximation, with *U*(H) set to 1.2*U*_eq_(C).

### Crystal data

**Table:**

[Fe(C_5_H_5_)(C_12_H_11_N_2_)]	*F*(000) = 632
*M**_r_* = 304.17	*D*_x_ = 1.449 Mg m^−^^3^
Orthorhombic, *P**c**a*2_1_	Mo *K*α radiation, λ = 0.71069 Å
Hall symbol: P 2c -2ac	Cell parameters from 3041 reflections
*a* = 18.7988 (17) Å	θ = 2.7–27.9°
*b* = 5.9314 (6) Å	µ = 1.07 mm^−^^1^
*c* = 12.5083 (10) Å	*T* = 200 K
*V* = 1394.7 (2) Å^3^	Platelet, red
*Z* = 4	0.32 × 0.18 × 0.13 mm

### Data collection

**Table:**

Bruker APEXII CCD diffractometer	2664 reflections with *I* > 2σ(*I*)
Radiation source: fine-focus sealed tube	*R*_int_ = 0.059
graphite	θ_max_ = 28.3°, θ_min_ = 2.7°
φ and ω scans	*h* = −24→18
6850 measured reflections	*k* = −7→4
3226 independent reflections	*l* = −16→16

### Refinement

**Table:**

Refinement on *F*^2^	Secondary atom site location: difference Fourier map
Least-squares matrix: full	Hydrogen site location: inferred from neighbouring sites
*R*[*F*^2^ > 2σ(*F*^2^)] = 0.033	H-atom parameters constrained
*wR*(*F*^2^) = 0.073	*w* = 1/[σ^2^(*F*_o_^2^) + (0.0358*P*)^2^] where *P* = (*F*_o_^2^ + 2*F*_c_^2^)/3
*S* = 0.94	(Δ/σ)_max_ < 0.001
3226 reflections	Δρ_max_ = 0.63 e Å^−^^3^
181 parameters	Δρ_min_ = −0.34 e Å^−^^3^
1 restraint	Absolute structure: Flack (1983), 1437 Friedel pairs
Primary atom site location: structure-invariant direct methods	Flack parameter: −0.010 (18)

### Fractional atomic coordinates and isotropic or equivalent isotropic displacement parameters (Å^2^)

**Table:**

	*x*	*y*	*z*	*U*_iso_*/*U*_eq_	
Fe1	0.429304 (15)	0.27995 (5)	0.40012 (4)	0.02708 (9)	
N1	0.29625 (12)	0.6770 (4)	0.24303 (18)	0.0351 (5)	
N2	0.14153 (15)	1.2176 (4)	0.3715 (2)	0.0511 (8)	
C1	0.31665 (13)	0.4737 (4)	0.24620 (19)	0.0325 (6)	
H1	0.2821	0.3579	0.2382	0.039*	
C2	0.21981 (15)	0.7154 (5)	0.2288 (2)	0.0404 (6)	
H2A	0.1945	0.5691	0.2290	0.048*	
H2B	0.2113	0.7889	0.1589	0.048*	
C11	0.39040 (16)	0.4099 (5)	0.2615 (2)	0.0304 (6)	
C12	0.44733 (14)	0.5451 (4)	0.2990 (2)	0.0316 (6)	
H12	0.4445	0.7007	0.3165	0.038*	
C13	0.50912 (14)	0.4093 (5)	0.3063 (2)	0.0375 (6)	
H13	0.5548	0.4578	0.3293	0.045*	
C14	0.49069 (16)	0.1873 (5)	0.2728 (2)	0.0372 (7)	
H14	0.5223	0.0626	0.2694	0.045*	
C15	0.41843 (16)	0.1830 (5)	0.2459 (2)	0.0355 (6)	
H15	0.3924	0.0554	0.2218	0.043*	
C21	0.34823 (18)	0.2684 (6)	0.5077 (3)	0.0535 (9)	
H21	0.3009	0.3187	0.4956	0.064*	
C22	0.4048 (2)	0.3982 (7)	0.5483 (3)	0.0539 (10)	
H22	0.4024	0.5524	0.5686	0.065*	
C23	0.4649 (2)	0.2613 (6)	0.5535 (2)	0.0529 (9)	
H23	0.5107	0.3065	0.5774	0.064*	
C24	0.44638 (19)	0.0467 (6)	0.5177 (2)	0.0480 (8)	
H24	0.4772	−0.0800	0.5139	0.058*	
C25	0.37477 (18)	0.0497 (5)	0.4883 (2)	0.0478 (8)	
H25	0.3485	−0.0737	0.4602	0.057*	
C31	0.19150 (14)	0.8618 (5)	0.3166 (2)	0.0334 (7)	
C32	0.16642 (16)	1.0750 (6)	0.2967 (2)	0.0396 (7)	
H32	0.1667	1.1256	0.2246	0.047*	
C33	0.14093 (19)	1.1409 (7)	0.4710 (3)	0.0488 (8)	
H33	0.1238	1.2377	0.5258	0.059*	
C34	0.16360 (18)	0.9303 (7)	0.4993 (2)	0.0496 (8)	
H34	0.1609	0.8818	0.5716	0.059*	
C35	0.19013 (17)	0.7907 (5)	0.4224 (2)	0.0440 (8)	
H35	0.2076	0.6457	0.4411	0.053*	

### Atomic displacement parameters (Å^2^)

**Table:**

	*U*^11^	*U*^22^	*U*^33^	*U*^12^	*U*^13^	*U*^23^
Fe1	0.03100 (16)	0.02611 (15)	0.02413 (13)	−0.00168 (13)	0.0003 (2)	0.0016 (3)
N1	0.0355 (12)	0.0351 (12)	0.0347 (12)	0.0009 (10)	0.0006 (9)	0.0047 (10)
N2	0.0590 (16)	0.0405 (15)	0.054 (2)	0.0138 (12)	−0.0009 (13)	0.0023 (13)
C1	0.0359 (14)	0.0358 (15)	0.0258 (11)	−0.0040 (11)	−0.0021 (10)	−0.0001 (11)
C2	0.0382 (15)	0.0449 (16)	0.0380 (14)	0.0041 (12)	−0.0071 (13)	−0.0035 (14)
C11	0.0406 (16)	0.0282 (16)	0.0223 (12)	0.0003 (13)	0.0008 (11)	0.0004 (12)
C12	0.0378 (15)	0.0287 (13)	0.0284 (12)	−0.0030 (11)	0.0035 (10)	0.0039 (11)
C13	0.0308 (14)	0.0460 (17)	0.0356 (13)	−0.0053 (12)	0.0037 (11)	0.0020 (13)
C14	0.0393 (17)	0.0414 (17)	0.0310 (14)	0.0112 (13)	0.0063 (12)	0.0008 (13)
C15	0.0461 (17)	0.0328 (16)	0.0277 (12)	−0.0025 (13)	−0.0001 (11)	−0.0029 (13)
C21	0.0463 (18)	0.074 (2)	0.0403 (15)	0.0117 (17)	0.0169 (15)	0.0221 (17)
C22	0.091 (3)	0.046 (2)	0.0253 (14)	0.001 (2)	0.0214 (17)	−0.0033 (17)
C23	0.060 (2)	0.070 (3)	0.0289 (15)	−0.008 (2)	−0.0063 (16)	0.0048 (16)
C24	0.065 (2)	0.0457 (18)	0.0332 (14)	0.0068 (16)	0.0004 (13)	0.0162 (14)
C25	0.056 (2)	0.0458 (17)	0.0410 (15)	−0.0161 (15)	0.0061 (14)	0.0094 (15)
C31	0.0249 (14)	0.0372 (16)	0.0382 (15)	−0.0017 (12)	−0.0054 (11)	0.0049 (13)
C32	0.0433 (16)	0.0383 (17)	0.0371 (15)	0.0000 (14)	−0.0029 (13)	0.0077 (16)
C33	0.050 (2)	0.056 (2)	0.0410 (16)	0.0080 (19)	0.0059 (14)	−0.0078 (18)
C34	0.055 (2)	0.062 (2)	0.0315 (15)	0.0040 (19)	−0.0001 (14)	0.0077 (18)
C35	0.0489 (16)	0.0421 (14)	0.041 (2)	0.0101 (13)	−0.0006 (12)	0.0110 (15)

### Geometric parameters (Å, °)

**Table:**

Fe1—C15	2.023 (3)	C13—C14	1.424 (4)
Fe1—C25	2.033 (3)	C13—H13	0.9500
Fe1—C11	2.034 (3)	C14—C15	1.400 (4)
Fe1—C21	2.034 (3)	C14—H14	0.9500
Fe1—C23	2.034 (3)	C15—H15	0.9500
Fe1—C22	2.034 (3)	C21—C22	1.408 (5)
Fe1—C14	2.042 (3)	C21—C25	1.411 (5)
Fe1—C24	2.044 (3)	C21—H21	0.9500
Fe1—C12	2.046 (2)	C22—C23	1.393 (6)
Fe1—C13	2.054 (3)	C22—H22	0.9500
N1—C1	1.266 (3)	C23—C24	1.394 (4)
N1—C2	1.466 (3)	C23—H23	0.9500
N2—C33	1.326 (5)	C24—C25	1.396 (5)
N2—C32	1.346 (4)	C24—H24	0.9500
C1—C11	1.450 (4)	C25—H25	0.9500
C1—H1	0.9500	C31—C32	1.373 (4)
C2—C31	1.498 (4)	C31—C35	1.389 (4)
C2—H2A	0.9900	C32—H32	0.9500
C2—H2B	0.9900	C33—C34	1.366 (5)
C11—C12	1.417 (4)	C33—H33	0.9500
C11—C15	1.458 (4)	C34—C35	1.363 (4)
C12—C13	1.416 (4)	C34—H34	0.9500
C12—H12	0.9500	C35—H35	0.9500
			
C15—Fe1—C25	105.98 (12)	C11—C12—H12	125.7
C15—Fe1—C11	42.13 (12)	Fe1—C12—H12	126.6
C25—Fe1—C11	122.39 (13)	C12—C13—C14	107.9 (2)
C15—Fe1—C21	123.05 (14)	C12—C13—Fe1	69.50 (15)
C25—Fe1—C21	40.60 (13)	C14—C13—Fe1	69.19 (16)
C11—Fe1—C21	107.90 (13)	C12—C13—H13	126.0
C15—Fe1—C23	156.44 (14)	C14—C13—H13	126.0
C25—Fe1—C23	67.53 (14)	Fe1—C13—H13	126.8
C11—Fe1—C23	160.53 (14)	C15—C14—C13	108.9 (3)
C21—Fe1—C23	67.72 (16)	C15—C14—Fe1	69.15 (15)
C15—Fe1—C22	160.68 (14)	C13—C14—Fe1	70.10 (15)
C25—Fe1—C22	67.87 (16)	C15—C14—H14	125.6
C11—Fe1—C22	124.38 (12)	C13—C14—H14	125.6
C21—Fe1—C22	40.49 (15)	Fe1—C14—H14	126.8
C23—Fe1—C22	40.03 (16)	C14—C15—C11	107.5 (3)
C15—Fe1—C14	40.28 (11)	C14—C15—Fe1	70.57 (15)
C25—Fe1—C14	121.82 (13)	C11—C15—Fe1	69.31 (14)
C11—Fe1—C14	68.91 (12)	C14—C15—H15	126.2
C21—Fe1—C14	158.47 (15)	C11—C15—H15	126.2
C23—Fe1—C14	122.33 (18)	Fe1—C15—H15	125.5
C22—Fe1—C14	158.61 (15)	C22—C21—C25	107.3 (3)
C15—Fe1—C24	120.60 (14)	C22—C21—Fe1	69.77 (18)
C25—Fe1—C24	40.03 (13)	C25—C21—Fe1	69.64 (17)
C11—Fe1—C24	157.84 (13)	C22—C21—H21	126.3
C21—Fe1—C24	67.61 (13)	C25—C21—H21	126.3
C23—Fe1—C24	39.96 (12)	Fe1—C21—H21	125.8
C22—Fe1—C24	67.29 (15)	C23—C22—C21	108.1 (3)
C14—Fe1—C24	106.85 (13)	C23—C22—Fe1	69.98 (18)
C15—Fe1—C12	69.27 (11)	C21—C22—Fe1	69.74 (19)
C25—Fe1—C12	159.25 (13)	C23—C22—H22	126.0
C11—Fe1—C12	40.66 (11)	C21—C22—H22	126.0
C21—Fe1—C12	123.97 (12)	Fe1—C22—H22	125.9
C23—Fe1—C12	124.75 (13)	C22—C23—C24	108.4 (4)
C22—Fe1—C12	109.60 (14)	C22—C23—Fe1	69.99 (19)
C14—Fe1—C12	68.36 (12)	C24—C23—Fe1	70.40 (18)
C24—Fe1—C12	159.82 (12)	C22—C23—H23	125.8
C15—Fe1—C13	68.60 (12)	C24—C23—H23	125.8
C25—Fe1—C13	158.36 (13)	Fe1—C23—H23	125.4
C11—Fe1—C13	68.51 (11)	C23—C24—C25	108.3 (3)
C21—Fe1—C13	159.72 (15)	C23—C24—Fe1	69.64 (17)
C23—Fe1—C13	108.61 (14)	C25—C24—Fe1	69.53 (17)
C22—Fe1—C13	123.86 (15)	C23—C24—H24	125.9
C14—Fe1—C13	40.70 (12)	C25—C24—H24	125.9
C24—Fe1—C13	123.32 (13)	Fe1—C24—H24	126.5
C12—Fe1—C13	40.41 (11)	C24—C25—C21	107.9 (3)
C1—N1—C2	116.7 (2)	C24—C25—Fe1	70.43 (17)
C33—N2—C32	116.1 (3)	C21—C25—Fe1	69.76 (17)
N1—C1—C11	122.9 (3)	C24—C25—H25	126.0
N1—C1—H1	118.6	C21—C25—H25	126.0
C11—C1—H1	118.6	Fe1—C25—H25	125.4
N1—C2—C31	110.4 (2)	C32—C31—C35	116.5 (3)
N1—C2—H2A	109.6	C32—C31—C2	121.5 (3)
C31—C2—H2A	109.6	C35—C31—C2	122.0 (3)
N1—C2—H2B	109.6	N2—C32—C31	124.9 (3)
C31—C2—H2B	109.6	N2—C32—H32	117.5
H2A—C2—H2B	108.1	C31—C32—H32	117.5
C12—C11—C1	128.2 (3)	N2—C33—C34	123.7 (3)
C12—C11—C15	107.1 (2)	N2—C33—H33	118.2
C1—C11—C15	124.7 (3)	C34—C33—H33	118.2
C12—C11—Fe1	70.14 (14)	C35—C34—C33	119.1 (3)
C1—C11—Fe1	123.72 (18)	C35—C34—H34	120.4
C15—C11—Fe1	68.56 (16)	C33—C34—H34	120.4
C13—C12—C11	108.6 (2)	C34—C35—C31	119.6 (3)
C13—C12—Fe1	70.09 (15)	C34—C35—H35	120.2
C11—C12—Fe1	69.20 (15)	C31—C35—H35	120.2
C13—C12—H12	125.7		
			
C2—N1—C1—C11	−179.0 (2)	C14—Fe1—C15—C11	−118.4 (3)
C1—N1—C2—C31	125.0 (3)	C24—Fe1—C15—C11	161.84 (18)
N1—C1—C11—C12	15.9 (4)	C12—Fe1—C15—C11	−37.75 (16)
N1—C1—C11—C15	−167.9 (3)	C13—Fe1—C15—C11	−81.19 (18)
N1—C1—C11—Fe1	106.2 (3)	C15—Fe1—C21—C22	−166.4 (2)
C15—Fe1—C11—C12	−118.5 (2)	C25—Fe1—C21—C22	118.4 (3)
C25—Fe1—C11—C12	163.94 (17)	C11—Fe1—C21—C22	−122.4 (2)
C21—Fe1—C11—C12	121.67 (19)	C23—Fe1—C21—C22	37.4 (2)
C23—Fe1—C11—C12	48.0 (5)	C14—Fe1—C21—C22	159.8 (4)
C22—Fe1—C11—C12	80.0 (2)	C24—Fe1—C21—C22	80.8 (2)
C14—Fe1—C11—C12	−80.94 (18)	C12—Fe1—C21—C22	−80.4 (2)
C24—Fe1—C11—C12	−163.9 (3)	C13—Fe1—C21—C22	−46.1 (5)
C13—Fe1—C11—C12	−37.11 (16)	C15—Fe1—C21—C25	75.2 (2)
C15—Fe1—C11—C1	118.2 (3)	C11—Fe1—C21—C25	119.2 (2)
C25—Fe1—C11—C1	40.6 (3)	C23—Fe1—C21—C25	−81.0 (2)
C21—Fe1—C11—C1	−1.6 (3)	C22—Fe1—C21—C25	−118.4 (3)
C23—Fe1—C11—C1	−75.3 (5)	C14—Fe1—C21—C25	41.4 (5)
C22—Fe1—C11—C1	−43.3 (3)	C24—Fe1—C21—C25	−37.6 (2)
C14—Fe1—C11—C1	155.8 (3)	C12—Fe1—C21—C25	161.19 (18)
C24—Fe1—C11—C1	72.8 (4)	C13—Fe1—C21—C25	−164.4 (3)
C12—Fe1—C11—C1	−123.3 (3)	C25—C21—C22—C23	0.1 (4)
C13—Fe1—C11—C1	−160.4 (3)	Fe1—C21—C22—C23	−59.7 (2)
C25—Fe1—C11—C15	−77.6 (2)	C25—C21—C22—Fe1	59.8 (2)
C21—Fe1—C11—C15	−119.8 (2)	C15—Fe1—C22—C23	155.8 (4)
C23—Fe1—C11—C15	166.5 (4)	C25—Fe1—C22—C23	80.9 (2)
C22—Fe1—C11—C15	−161.4 (2)	C11—Fe1—C22—C23	−164.1 (2)
C14—Fe1—C11—C15	37.58 (17)	C21—Fe1—C22—C23	119.1 (3)
C24—Fe1—C11—C15	−45.4 (4)	C14—Fe1—C22—C23	−40.6 (5)
C12—Fe1—C11—C15	118.5 (2)	C24—Fe1—C22—C23	37.5 (2)
C13—Fe1—C11—C15	81.41 (18)	C12—Fe1—C22—C23	−121.1 (2)
C1—C11—C12—C13	177.0 (2)	C13—Fe1—C22—C23	−78.4 (3)
C15—C11—C12—C13	0.4 (3)	C15—Fe1—C22—C21	36.7 (6)
Fe1—C11—C12—C13	59.20 (18)	C25—Fe1—C22—C21	−38.2 (2)
C1—C11—C12—Fe1	117.8 (3)	C11—Fe1—C22—C21	76.8 (2)
C15—C11—C12—Fe1	−58.83 (18)	C23—Fe1—C22—C21	−119.1 (3)
C15—Fe1—C12—C13	−80.94 (17)	C14—Fe1—C22—C21	−159.7 (3)
C25—Fe1—C12—C13	−161.3 (3)	C24—Fe1—C22—C21	−81.6 (2)
C11—Fe1—C12—C13	−120.0 (2)	C12—Fe1—C22—C21	119.8 (2)
C21—Fe1—C12—C13	162.43 (19)	C13—Fe1—C22—C21	162.51 (19)
C23—Fe1—C12—C13	77.5 (2)	C21—C22—C23—C24	−0.6 (4)
C22—Fe1—C12—C13	119.62 (19)	Fe1—C22—C23—C24	−60.2 (2)
C14—Fe1—C12—C13	−37.61 (16)	C21—C22—C23—Fe1	59.6 (2)
C24—Fe1—C12—C13	42.3 (4)	C15—Fe1—C23—C22	−160.1 (3)
C15—Fe1—C12—C11	39.07 (17)	C25—Fe1—C23—C22	−81.9 (3)
C25—Fe1—C12—C11	−41.2 (4)	C11—Fe1—C23—C22	42.8 (6)
C21—Fe1—C12—C11	−77.6 (2)	C21—Fe1—C23—C22	−37.8 (2)
C23—Fe1—C12—C11	−162.4 (2)	C14—Fe1—C23—C22	163.7 (2)
C22—Fe1—C12—C11	−120.37 (19)	C24—Fe1—C23—C22	−119.1 (3)
C14—Fe1—C12—C11	82.40 (19)	C12—Fe1—C23—C22	79.0 (3)
C24—Fe1—C12—C11	162.3 (3)	C13—Fe1—C23—C22	120.9 (2)
C13—Fe1—C12—C11	120.0 (2)	C15—Fe1—C23—C24	−41.1 (5)
C11—C12—C13—C14	0.1 (3)	C25—Fe1—C23—C24	37.2 (2)
Fe1—C12—C13—C14	58.71 (19)	C11—Fe1—C23—C24	161.9 (4)
C11—C12—C13—Fe1	−58.65 (17)	C21—Fe1—C23—C24	81.3 (2)
C15—Fe1—C13—C12	82.76 (17)	C22—Fe1—C23—C24	119.1 (3)
C25—Fe1—C13—C12	162.0 (3)	C14—Fe1—C23—C24	−77.2 (2)
C11—Fe1—C13—C12	37.33 (16)	C12—Fe1—C23—C24	−161.95 (19)
C21—Fe1—C13—C12	−46.2 (4)	C13—Fe1—C23—C24	−120.0 (2)
C23—Fe1—C13—C12	−122.16 (18)	C22—C23—C24—C25	0.9 (4)
C22—Fe1—C13—C12	−80.5 (2)	Fe1—C23—C24—C25	−59.0 (2)
C14—Fe1—C13—C12	119.6 (2)	C22—C23—C24—Fe1	59.9 (2)
C24—Fe1—C13—C12	−163.87 (17)	C15—Fe1—C24—C23	162.2 (2)
C15—Fe1—C13—C14	−36.79 (17)	C25—Fe1—C24—C23	−119.7 (3)
C25—Fe1—C13—C14	42.5 (4)	C11—Fe1—C24—C23	−164.1 (3)
C11—Fe1—C13—C14	−82.22 (19)	C21—Fe1—C24—C23	−81.6 (3)
C21—Fe1—C13—C14	−165.8 (4)	C22—Fe1—C24—C23	−37.5 (2)
C23—Fe1—C13—C14	118.3 (2)	C14—Fe1—C24—C23	120.6 (2)
C22—Fe1—C13—C14	160.0 (2)	C12—Fe1—C24—C23	47.6 (5)
C24—Fe1—C13—C14	76.6 (2)	C13—Fe1—C24—C23	79.1 (3)
C12—Fe1—C13—C14	−119.6 (2)	C15—Fe1—C24—C25	−78.1 (2)
C12—C13—C14—C15	−0.5 (3)	C11—Fe1—C24—C25	−44.4 (4)
Fe1—C13—C14—C15	58.41 (19)	C21—Fe1—C24—C25	38.2 (2)
C12—C13—C14—Fe1	−58.90 (18)	C23—Fe1—C24—C25	119.7 (3)
C25—Fe1—C14—C15	76.6 (2)	C22—Fe1—C24—C25	82.2 (2)
C11—Fe1—C14—C15	−39.25 (19)	C14—Fe1—C24—C25	−119.7 (2)
C21—Fe1—C14—C15	46.2 (4)	C12—Fe1—C24—C25	167.3 (3)
C23—Fe1—C14—C15	158.6 (2)	C13—Fe1—C24—C25	−161.25 (18)
C22—Fe1—C14—C15	−171.7 (4)	C23—C24—C25—C21	−0.9 (3)
C24—Fe1—C14—C15	117.7 (2)	Fe1—C24—C25—C21	−59.9 (2)
C12—Fe1—C14—C15	−83.06 (19)	C23—C24—C25—Fe1	59.0 (2)
C13—Fe1—C14—C15	−120.4 (3)	C22—C21—C25—C24	0.5 (4)
C15—Fe1—C14—C13	120.4 (3)	Fe1—C21—C25—C24	60.4 (2)
C25—Fe1—C14—C13	−162.96 (18)	C22—C21—C25—Fe1	−59.9 (2)
C11—Fe1—C14—C13	81.16 (18)	C15—Fe1—C25—C24	118.8 (2)
C21—Fe1—C14—C13	166.6 (3)	C11—Fe1—C25—C24	161.80 (19)
C23—Fe1—C14—C13	−81.0 (2)	C21—Fe1—C25—C24	−118.6 (3)
C22—Fe1—C14—C13	−51.3 (5)	C23—Fe1—C25—C24	−37.1 (2)
C24—Fe1—C14—C13	−121.87 (18)	C22—Fe1—C25—C24	−80.6 (2)
C12—Fe1—C14—C13	37.35 (16)	C14—Fe1—C25—C24	78.0 (2)
C13—C14—C15—C11	0.7 (3)	C12—Fe1—C25—C24	−167.6 (3)
Fe1—C14—C15—C11	59.71 (18)	C13—Fe1—C25—C24	46.8 (4)
C13—C14—C15—Fe1	−58.99 (19)	C15—Fe1—C25—C21	−122.5 (2)
C12—C11—C15—C14	−0.7 (3)	C11—Fe1—C25—C21	−79.6 (2)
C1—C11—C15—C14	−177.5 (2)	C23—Fe1—C25—C21	81.5 (2)
Fe1—C11—C15—C14	−60.50 (19)	C22—Fe1—C25—C21	38.1 (2)
C12—C11—C15—Fe1	59.83 (17)	C14—Fe1—C25—C21	−163.4 (2)
C1—C11—C15—Fe1	−117.0 (2)	C24—Fe1—C25—C21	118.6 (3)
C25—Fe1—C15—C14	−120.7 (2)	C12—Fe1—C25—C21	−49.0 (4)
C11—Fe1—C15—C14	118.4 (3)	C13—Fe1—C25—C21	165.4 (3)
C21—Fe1—C15—C14	−161.6 (2)	N1—C2—C31—C32	113.6 (3)
C23—Fe1—C15—C14	−50.5 (5)	N1—C2—C31—C35	−65.5 (4)
C22—Fe1—C15—C14	170.9 (4)	C33—N2—C32—C31	−0.9 (5)
C24—Fe1—C15—C14	−79.8 (2)	C35—C31—C32—N2	0.5 (5)
C12—Fe1—C15—C14	80.61 (19)	C2—C31—C32—N2	−178.6 (3)
C13—Fe1—C15—C14	37.16 (19)	C32—N2—C33—C34	−0.2 (5)
C25—Fe1—C15—C11	120.95 (18)	N2—C33—C34—C35	1.7 (6)
C21—Fe1—C15—C11	80.1 (2)	C33—C34—C35—C31	−2.1 (5)
C23—Fe1—C15—C11	−168.8 (3)	C32—C31—C35—C34	1.1 (4)
C22—Fe1—C15—C11	52.5 (5)	C2—C31—C35—C34	−179.8 (3)

### Hydrogen-bond geometry (Å, °)

**Table:**

Cg is the centroid of the C21–C25 ring.

**Table:**

*D*—H···*A*	*D*—H	H···*A*	*D*···*A*	*D*—H···*A*
C13—H13···N2^i^	0.95	2.58	3.429 (4)	149
C34—H34···N1^ii^	0.95	2.59	3.482 (4)	156
C32—H32···Cg^iii^	0.95	2.92	3.775 (3)	150

Symmetry codes: (i) *x*+1/2, −*y*+2, *z*; (ii) −*x*+1/2, *y*, *z*+1/2; (iii) −*x*+1/2, *y*+1, *z*−1/2.
